# Internet Behavior and Satisfaction With Sleep, Health, Quality of Life and Physical Activity Self-Efficacy as Components of Subjective Well-Being: Findings From an Online Survey

**DOI:** 10.5964/ejop.5343

**Published:** 2022-11-30

**Authors:** Endi Guza, Lingling Gao, Sonia Lippke

**Affiliations:** 1 Faculty of Psychology & Speech Therapy, University of Valencia, Valencia, Spain; 2 Department of Psychology & Methods, Focus Area Diversity, Constructor University Bremen (Previouly known as Jacobs University Bremen), Bremen, Germany; Trinity College Dublin, Dublin, Ireland

**Keywords:** subjective well-being, physical activity, internet use, health-related apps, online behavior

## Abstract

This study aimed to examine the relationship between internet use (constructive and health-related internet behavior, health app usages), physical activity self-efficacy, and subjective well-being (quality of life, health satisfaction, sleep satisfaction). Participants (N = 758) were recruited to participate in an online survey. One-way MANOVA and multiple regression analyses were used to examine the hypotheses. Results showed that internet use was negatively associated with sleep satisfaction, r(738) = -.127, p < .001. Individuals who use health-related apps for movement/fitness, t(689.900) = -3.354, p < .001, nutrition, t(300.075) = -2.434, p = .016, information for self-diagnosis, t(199.768) = -2.321, p = .021, and contact with doctors, t(90.630) = -2.035, p = .045, have higher PA self-efficacy than those who do not. Overall, there was a statistically significant difference in subjective well-being based on a participants’ constructive internet use, F(28, 2590) = 1.97, p = .002, with quality of life (p = .006) and sleep satisfaction (p = .025) being statistically significant components of subjective well-being. This paper discusses the important theoretical and practical implications regarding the development of health-related apps and online well-being interventions which are significantly relevant to the well-being literature.

The search for the secret to a good life has persisted since ancient times, spurred on by Greek philosophers and religious doctrines, until it recently became established as a scientific endeavor as the study of well-being. To date, Diener’s *tripartite model of subjective well-being* remains the most relevant and influential self-reported measure of well-being still used in scientific research. His model of subjective well-being consists of three components: positive affect, negative affect, and life satisfaction (Diener, 1994). These components are distinct from, yet interrelated with each other, as people who have higher levels of satisfaction with life tend to experience positive emotions more frequently than negative ones. Research has shown that well-being is linked to several factors implemented in health and well-being interventions, including internet-based interventions. This study aims to assess whether internet behavior is related to subjective well-being, while examining its relevant components. In this study, internet behavior refers to constructive internet use and health-related internet use. Constructive internet use refers to the frequency of participants using the internet for work or study purposes, while health-related internet use refers specifically to the use of health apps. Since working and studying are usually behaviors repeated on a regular basis, constructive internet use was measured in terms of days per week.

## Subjective Well-Being and Its Components

The concept of “subjective” well-being implies that people can evaluate their own lives and experiences meaningfully (Tov & Diener, 2013), an assumption validated by many studies analyzing subjective well-being and its components such as sleep quality (Weinberg et al., 2016), social relationships (Hitchcott et al., 2017), and personal growth (Negovan, 2010). Perceived physical health is a particularly important component of subjective well-being (Balázs et al., 2018). Røysamb et al. (2003) concluded that perceived health is a more significant component of subjective well-being than the current health status. They further concluded that this finding may be explained by genetic and environmental factors, as the genes which affect perceived health are those which affect subjective well-being.

Steptoe et al. (2015) assessed the relationship between subjective well-being and various health behaviors and indicators and concluded that amid lifestyle factors, physical activity (PA) was the most important mediator of the relationship between subjective well-being (specifically positive affect) and health, and is also linked to developmental, physical, mental, and social well-being (Stathi et al., 2002).

Moreover, PA is found to have a particularly beneficial impact on sleep quality—another influential factor underlying subjective well-being. Indeed, sleep quality and its components (such as frequency of awakenings at night and general satisfaction with sleep) have a greater influence on subjective well-being and overall measures of health compared to sleep quantity (Pilcher et al., 1997). This can be explained by the fact that brain functions control emotion regulation and restore homeostasis during periods of sleep (Weinberg et al., 2016).

## Well-Being-Related Internet Behaviors

Since its emergence, one of the primary aims of research on well-being has been to identify variables and ways to increase general well-being and overall satisfaction with life, with self-reported well-being measures being the most common method of assessment to collect empirical data. Consequently, numerous programs and intervention plans have been developed for this purpose. While online health interventions are continuously more popular, research on their effectiveness remains scarce. The internet as a medium carries the advantages of both an interpersonal and mass communication system. As such, it can be a very successful channel for improving well-being and stimulating health-related behavior change (Cassell et al., 1998).

Individuals are generally motivated to engage in behaviors that will provide them with positive outcome experiences or personal gratifications. Hobfoll’s conservation of resources theory predicts that people seek to create and maintain resources (e.g., personal characteristics, social circumstances, well-being, etc.) that they value, and avoid situations that risk the loss of such resources (Hobfoll, 1989). The more resources one has, the more likely one is to gain further resources. In other words, existing resource gains generate future gains, thus creating a gain spiral (Hobfoll, 1989). The conservation of resources theory can be applied to public health promotion strategies to increase their effectiveness. Hobfoll and Schumm (2009) conducted several controlled clinical trials where they highlighted the relevance of resource-driven improvement in psychological health promotion for stimulating individuals to engage in healthy sexual behaviors. These interventions provided individuals with the necessary personal, social, and physical resources to engage in safer sexual behaviors. Hobfoll and Schumm’s results revealed the multifaceted intervention to be highly effective. They further concluded that if health promotion strategies focused on increasing knowledge, motivation, and behavioral resources for enacting and maintaining healthy behaviors, the success rate would be significantly higher (Hobfoll & Schumm, 2009). Kim et al. (2015) also provided an explanation for the effectiveness of wellness programs from conservation of resources perspective. Based on their findings, they claim that wellness programs trigger a resource gain spiral by increasing one’s wellness self-efficacy to improve aspects of one’s well-being. Well-being in turn leads to increased psychological availability and career satisfaction over time (Kim et al., 2015). These findings were supported by Larose et al. (2001), who claimed that people tend to seek out media in a goal-directed manner that will provide them with personal gratification and the means to satisfy a wide variety of needs.

The conservation of resources theory (Hobfoll, 1989) can thus serve as a framework to rationalize the effectiveness of the internet as a platform for well-being interventions. Specifically, if online interventions provide individuals with resources to engage in healthy behaviors that increase their well-being, this creates a gain spiral which, in turn, leads people to engage in these behaviors more frequently and consistently. Such resources can be information on their health status (e.g., PA and sleep trackers, heart rate and blood pressure measurements, etc.), and guidance for reaching desired health goals, among others. In comparison to offline interventions, digital interventions have greater reach while being time-effective and customizable according to one’s individual needs. Empirical data shows that overall well-being interventions can be highly effective at improving subjective well-being and at promoting the achievement and maintenance of personal health goals (e.g., Lennefer et al., 2019; Mitchell et al., 2009).

## Health-Related App Usages and Subjective Well-Being

For online interventions to be successful, they must identify and implement effective behavior change techniques and strategies, such as behavior monitoring, problem identification and problem-solving, incentives for adopting healthy behaviors, and increasing self-efficacy. According to Mitchell et al. (2009), combining techniques from positive psychology and well-being research with online intervention research can significantly increase the effectiveness of health promotion. Many of the limitations of traditional offline interventions can now be tackled by online interventions, as the internet’s approach to health promotion has higher accessibility, sustainability, and personalization according to one’s needs (Mitchell et al., 2009) and provides social support digitally (Lippke et al., 2021). Scientific evidence shows that internet-delivered programs have a high success rate in many behavior change fields such as weight management, nutrition, smoking cessation, stress reduction, blood glucose control, reducing alcohol consumption, and increasing the level of PA (Carr et al., 2013).

A technique commonly used by many people to deepen their self-knowledge about health and well-being is tracking their individual behavior. Whether written down on paper or mentally memorized, many people keep track of their sleep and eating patterns, weight, diets, and exercise schedules. Lennefer et al. (2019) developed an online well-being intervention that combined a behavioral and cognitive approach. The intervention aimed to increase intrinsic and extrinsic motivation in employees through enhancing enjoyment levels, setting personal goals, and receiving personalized advice from online coaches. Findings indicated that this design was used by participants one year after the trial intervention and was highly effective at increasing employees’ health perception and reducing their body mass index (BMI) with activity trackers positively impacting subjective well-being.

While research on internet-based interventions has persisted for the past two decades, the recent proliferation of smartphone applications (apps) has led to the development of health-related apps which include monitoring of health indicators, personal and public health information, access to clinical health data and medical advice, and allow for communication with health workers. Health apps are highly practical to use as they are easily accessible through one’s smartphone or smartwatch and can be synchronized to one’s schedule or other daily activities.

Yuan et al. (2015) assessed users’ perception of health apps and found that performance expectancy (i.e., the extent to which users will gain benefits from using the app), combined with fun and engaging features, were among the main predictors of users’ continued use of apps. Moreover, they found that apps which facilitated habitual use were highly effective, as falling into regular routines led to task completion without little conscious thought (Yuan et al., 2015). This finding is particularly relevant for health apps designed to manage habitual health behaviors like PA, dieting, nutrition, and smoking cessation. Personalized information, reliable and automatic tracking of healthy behaviors, goal-setting features, and daily reminders were also among the most desirable features for health apps (Peng et al., 2016).

## Research Question and Hypotheses

Many efforts have been made to explore the association between internet behavior and well-being, however, less is known about whether constructive and health-related internet behavior is related to individual’s subjective well-being. The research question of this study was whether internet behavior is related to subjective well-being, while examining its relevant components. To answer the research question, the following *hypotheses* were assessed:

1. Constructive internet use will be positively correlated with sleep satisfaction and quality of life.
2. People who use health-related apps will have a higher quality of life compared to non-users.
3. People who use health-related apps will have higher PA self-efficacy compared to non-users.
4. Constructive internet use will be positively correlated with subjective well-being.
5. Constructive internet use and health-related internet use will significantly predict quality of life, health satisfaction, sleep satisfaction, and PA self-efficacy.

These hypotheses were examined in an online survey, where participants self-reported their intentions and frequency of constructive internet use, health-related internet use, as well as various aspects of their subjective well-being.

## Method

### Participants

The online questionnaire gathered 1,375 respondents in total. After excluding invalid data, 758 participants were included in the current study. Among the participants, 60.9% were female and the mean age was 27.6 years old (*SD* = 13.25). The majority of the participants were either students or employed (93.9%), out of which 44.6% were employees or students at Jacobs University Bremen (now Constructor University Bremen).

### Procedure

The data was collected from October 2016 to August 2018 through online questionnaires, which consisted of both multiple-choice and open-ended questions. In Germany, the questionnaires were distributed to university staff and students, who received both an English and German version of the questionnaire through email, Facebook, and face-to-face link distribution. In China, the survey link was sent to students and staff at universities, and was distributed to residents of Shijiazhuang, China. Participants in China received the Chinese version through email and face-to-face link distribution.

### Measurements

Internet behavior was assessed in terms of constructive internet use and health-related internet use. Constructive internet use was measured by the item “*How many days did you spend on internet use for work/study purposes per week*?” Participants could only fill in numbers. To measure health-related internet use participants were asked “*For which topics based around health do you use apps*?” The participants could select multiple answers among the following options:

“*Movement or fitness (e.g., Pedometer)”*
*“Relaxation (e.g., Yoga)”*
*“Nutrition”*
*“Weight loss (e.g., Counting calories)”*
*“Measuring sleeping habits”*
*“Smoking cessation”*
*“Blood pressure and heart frequency measurement”*
*“Information for self-diagnosis”*
*“Medication intake”*
*“Contact with a doctor”*

Subjective well-being was measured in terms of satisfaction with health and sleep, quality of life, and PA self-efficacy. Satisfaction with health and sleep were measured by the items: “*How satisfied are you with your health?*”, and “*How satisfied are you with your sleep?*”, where answers were provided on a scale from 1 (Very dissatisfied) to 5 (Very satisfied). Quality of life was measured by the item “*How would you rate your quality of life?*” where answers were provided on a scale from 1 (Very poor) to 5 (Very good). These three one-item measures were adapted from the short version of the World Health Organization Quality of Life Questionnaire (WHO, 1995) and have been examined in previous studies (Hsiao et al., 2014; Lu et al., 2011). PA self-efficacy was measured by the item “*I feel certain that I can be physically active even if it is difficult*” where answers were provided on a scale from 1 (Completely disagree) to 4 (Completely agree). This one-item measure was used previously (Lippke et al., 2021). In addition, participants were asked demographic questions including sex, education level, work status, and whether they were employees or students at Jacobs University Bremen (now Constructor University Bremen). The questionnaires were translated by researchers in the psychological area. A research assistant proficient in German, English, and Chinese back-translated and checked all three language versions. One of the coauthors who is a native German speaker assessed the English and German versions of the questionnaires; another coauthor who is a native Chinese speaker assessed the English and Chinese versions.

### Data Analysis

The statistical analyses for this study were completed using IBM SPSS Statistics 24.0. To assess the first hypothesis, we conducted a bivariate Pearson correlation for the variables internet use for work/study, quality of life, and satisfaction with sleep. When analyzing health-related internet behavior, we treated participants’ use of each health-based app as a variable. Since health app use was assessed as a categorical variable with two levels (0—not using health app; 1—using health app), we conducted an independent samples *t*-test to assess the second and third hypotheses.

To assess the fourth hypothesis, we conducted a one-way MANOVA analysis, with the frequency of internet use for work/study as a fixed factor, and satisfaction with health, satisfaction with sleep, quality of life, and PA self-efficacy as dependent variables and components of subjective well-being. The MANOVA analysis can assess patterns between multiple dependent variables which tests with a single dependent variable can fail to detect. To assess the fifth hypothesis, we conducted four separate multiple-regression analyses. These tested whether internet use for work/study and use of health apps may predict quality of life in the first analysis, health satisfaction in the second analysis, sleep satisfaction in the third analysis, and PA self-efficacy in the fourth analysis.

## Results

### Descriptive Analyses

Among the components of subjective well-being rated by the participants (Table 1), quality of life was the highest-rated variable whereas sleep satisfaction was the lowest-rated.

**Table 1:** Descriptive Statistics for Components of Subjective Well-Being, and Intercorrelations

Variable	*N*	Range	*M*	*SD*	1	2	3	4
0. PA Self-Efficacy	747	1-4	3.33	0.846	.112**			
1. Quality of Life	753	1-5	3.59	0.936	1		
2. Health Satisfaction	752	1-5	3.29	1.053	.549**	1	
3. Sleep Satisfaction	740	1-5	2.99	1.178	.436	.427**	1
4. Internet Behavior	758	1-7	5.53	1.833	.055	-.025	-.127**	1

*Note*. *N* = Number of valid observations. *M* = Mean. *SD* = Standard deviation.

*p ≤ .05. **p ≤ .01. ***p ≤ .001.

**Figure 1:**
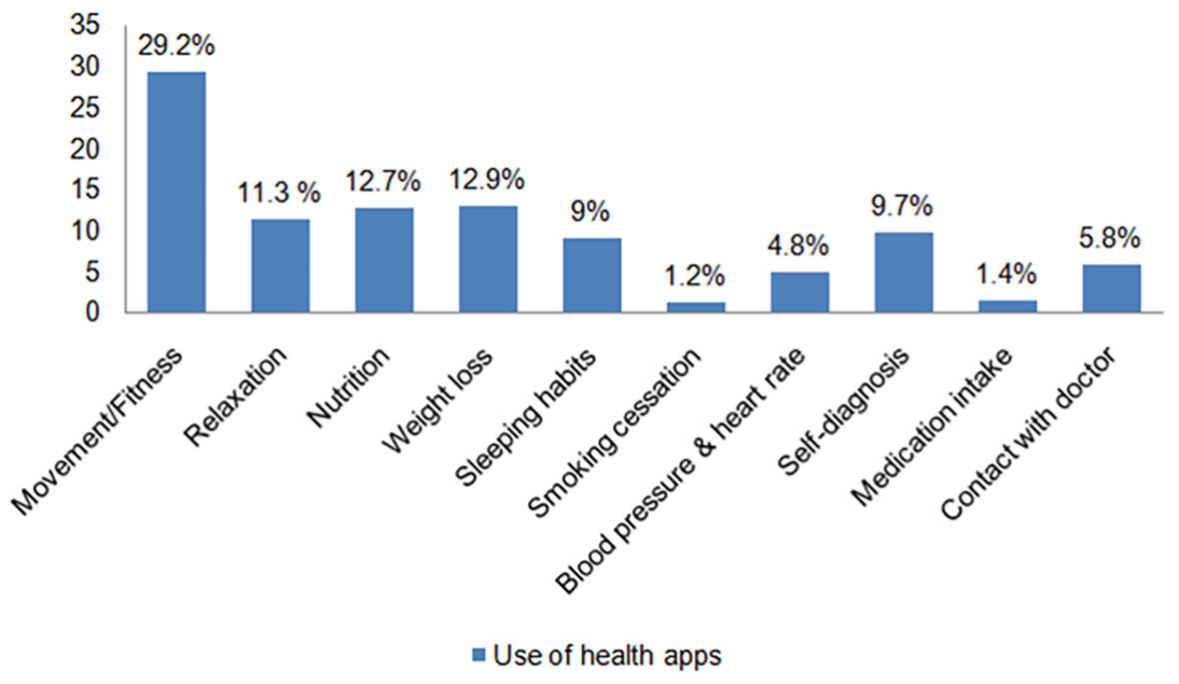
Percentage of App Users for Each Health-Related App *Note*. Multiple answers could be given by each participant for the use of health apps.

On average, the internet was used for work/study purposes 5.53 days per week (*SD* = 1.833). Participants’ use of each health-related app is presented in Figure 1. Apps for movement/fitness were used the most used among participants (29.2%), followed by apps for weight loss (12.9%) and nutrition (12.7%).

### Correlation Analyses

The bivariate Pearson correlation analysis found no significant correlation between the frequency of internet use for work/study, satisfaction with health, and PA self-efficacy. However, the frequency of internet use for work/study was negatively correlated with sleep satisfaction *r*(738) = -.127, *p* < .001. This suggests that study participants who use the internet constructively more often reported lower levels of satisfaction with sleep. However, no interrelation between internet use and quality of life was revealed. Thus, the first hypothesis was not supported by the data.

### Group Differences

To test Hypothesis 2 which stated that people who use health-related apps will have a higher quality of life compared to non-users, independent samples *t*-test were performed. No significant differences were found for the reported levels of quality of life (see Supplementary Materials section for Table A1); thus, the second hypothesis was rejected.

The independent samples *t*-test revealed that people who use apps for movement/fitness, nutrition, information for self-diagnosis, and contact with doctors have higher PA self-efficacy than those who do not (Table 2).

**Table 2:** Independent Samples t-Test Results: Comparing Levels of PA Self-Efficacy Among App Users and Non-Users

	App users		Non-users					
Variable	*M*	*SD*	*M*	*SD*	*t*	*df*	*p*	Cohen’s *d*
Apps for movement/fitness	3.43	0.735	3.22	0.937	-3.354	689.900	.001	.249
Apps for nutrition	3.46	0.725	3.30	0.873	-2.434	300.075	.016	.199
Apps for self-diagnosis	3.47	0.696	3.30	0.869	-2.321	199.768	.021	.216
Apps for contact with doctors	3.50	0.717	3.31	0.856	-2.035	90.630	.045	.240

*Note*. *M* = Mean. *SD* = Standard deviation. *df* = Degrees of freedom.

Among these measures, users of apps for contact with doctors reported the largest mean of PA self-efficacy (*M* = 3.50, *SD* = 0.717). However, no significant differences between users and non-users in PA self-efficacy were found for the use of apps for relaxation, weight loss, measuring sleeping habits, blood pressure and heart frequency measurement, smoking cessation, and medication intake (see Supplementary Materials section for Table A2). Therefore, the third hypothesis was only partially supported by the data.

To test Hypothesis 4, a one-way MANOVA analysis, Wilk’s Lambda multivariate test was run. The results proved that the effect of constructive internet use was positive at a significance level of *p* < .05 and accounted for 1.9% of subjective well-being (*F*(28, 2590) = 1.97, *p* = .002; partial η^2^ = .019). The tests of between-subjects effects showed that constructive internet use had a significant positive relationship with quality of life, *F*(7, 721) = 2.86, *p* = .006, partial η^2^ = .027, and sleep satisfaction, *F*(7, 721) = 2.31, *p* = .025, partial η^2^ = .022, while no relationship was found with satisfaction with health and PA self-efficacy. These results suggest that overall constructive internet use has a significant positive relationship with subjective well-being, with the components “quality of life” and “satisfaction with sleep” being statistically significant. The fourth hypothesis was therefore supported by the data.

To test Hypothesis 5, multiple regression analyses were performed. The results showed no significant association between constructive internet use and health apps use with quality of life and health satisfaction. However, constructive internet use and use of health apps’ significantly predicted satisfaction with sleep *F*(11, 728) = 2.36, *p* = .007, *R*^2^ = .034, where the frequency of internet use for work/study (β = -.127, *p *< .001) and apps for relaxation (β = -.076, *p* = .05) were significant predictors. The independent variables explained 3.4% of the variability of satisfaction with sleep. The negative β coefficients indicate that satisfaction with sleep is negatively correlated with predictors of internet use for work/study and apps for relaxation. PA self-efficacy was also significantly predicted by constructive internet use and health apps’ use *F*(11, 735) = 2.46, *p* = .005, R^2^ = .035; with apps for movement/fitness (β = .122, *p* = .002) and apps for relaxation (β = -.085, *p* = .029) as significant predictors. The independent variables explained 3.5% of the variability in PA self-efficacy, which is positively correlated with app usage for movement/fitness and negatively correlated to the use of apps for relaxation. Thus, Hypothesis 5 was partially supported by the data.

## Discussion

This study investigated the relationship between internet behavior and subjective well-being. Taken together, the results revealed that constructive internet use had a significant positive relationship with subjective well-being, with quality of life and sleep satisfaction being statistically significant components. Moreover, sleep satisfaction was predicted by both constructive and health-related internet use.

The first hypothesis, which examined whether individuals who engaged with more constructive internet use were more likely to have higher levels of satisfaction with sleep and quality of life, was not supported by the data. The results of the correlation analysis showed no significant association between constructive internet use and quality of life. A possible explanation for the lack of correlation in the current study could be that quality of life measure may be mediated by several other factors that were not assessed in this study, such as optimism and perceived support (Yalçın, 2011), working and living conditions, self-perceived stress (Rusli et al., 2008), as well as personality factors (Xie et al., 2016).

Contrary to the first hypothesis assumption, however, constructive internet use was negatively correlated with satisfaction with sleep. A possible explanation for this finding could be that people who use the internet for work and/or study purposes may engage in more extensive internet use as part of their professional or academic routine, which delays their sleep schedule. This relationship may also be mediated by other factors such as stress levels, health-related problems, and lifestyle approaches. Moreover, a recent study from Billari et al. (2018) found that increased use of the internet before sleep is strongly correlated to sleep deprivation, and that excessive internet use in general can significantly disturb sleeping habits.

The second hypothesis, which explored whether people engaged with increased health-related internet use were more likely to have a higher quality of life, was not supported by the data. The results of the *t*-test showed no significant differences in quality of life for people who use health apps and those who do not. Such results could be explained by the fact that the *t*-test only focuses on mean differences rather than a correlation itself. Moreover, the reason why individuals use health apps could be an influential mediator, as people who have certain health impairments may expect to have more prominent outcomes from using health apps. Choi et al. (2015) argues that using health apps has a positive impact on medication adherence and quality of life, which leads to higher effectiveness of medication therapy. Those without health impairments, however, might use health apps only for self-monitoring purposes and thus do not expect any outcome related to the quality of life. For example, a study conducted by Liang and Ploderer (2016) found that using health apps for sleep-tracking had a great impact on one’s self-knowledge about their sleeping patterns. However, it did not improve their sleep quality. Overall, the results suggest that using apps for informational and self-monitoring purposes might improve one’s level of self-awareness about health indicators, but there is no clear empirical evidence about their effect on one’s quality of life.

The third hypothesis, which examined whether people engaged with higher levels of health-related internet use were more likely to have higher levels of PA self-efficacy, was partially supported by the data. The results showed significant differences in PA self-efficacy in those who used only some of the health apps, especially apps for movement/fitness, nutrition, information for self-diagnosis, and contact with doctors. The relationship between using health apps for movement/fitness and nutrition and PA self-efficacy was expected, as it is implied that individuals who used these apps were more likely to be committed to fitness and PA. These findings can be explained through the conservation of resources theory whereby higher levels of engagement with PA results in higher levels of self-efficacy, which in turn increases engagement with PA. Initial efforts put into PA would create again a spiral that is further stimulated by increased PA self-efficacy.

Research from Litman et al (2015) shows that self-efficacy is a crucial factor mediating the relationship between the use of exercise apps and health outcomes. This argument suggests that app usage may increase PA levels, which in turn increases PA self-efficacy; whereas in comparison, app usage may increase PA self-efficacy, subsequently increasing PA levels, resulting in better health outcomes. Therefore, PA self-efficacy is both predicted by, and a predictor of PA (Litman et al., 2015). The link between PA self-efficacy and apps for information for self-diagnosis and contact with doctors may also be explained by individual health and app use choices. Apps for movement/fitness were the most used among participants (29.2%). As such, individuals who use these apps, or simply people who engage in PA, might also use apps for information for self-diagnosis and contact with doctors. Similarly, the lack of engagement in PA could explain the lack of significant differences in PA self-efficacy among the use of other health apps.

The fourth hypothesis, which examined whether people who engaged with higher levels of constructive internet use were more likely to have higher levels of subjective well-being, was supported by the data. The MANOVA results reported that overall, constructive internet use has a significant effect on subjective well-being, with quality of life and satisfaction with sleep emerging as the only statistically significant components. Regarding quality of life, the findings underscore findings by Ernsting et al. (2017) who, as mentioned previously, concluded that people with higher education and full employment are more likely to engage in constructive internet use and report having a better quality of life. The positive correlation between constructive internet use and quality of life may also be interpreted from conservation of resources perspective (Hobfoll, 1989) as those who engage in constructive internet use for work or study purposes may trigger a gain spiral, where they receive professional or academic gratification for their work, resulting in greater engagement with constructive internet use. This in turn may increase their level of perceived quality of life and lead to higher levels of subjective well-being.

Oravec (2002) argues that constructive online recreation can have a positive impact on creativity and well-being in the workplace. Wang et al. (2012) also found that using the internet for constructive purposes predicts healthy lifestyles among internet users. As the MANOVA analysis detects patterns between multiple dependent variables, which a simple correlational test may fail to detect, this may explain the lack of correlation found when assessing the first hypothesis. The relationship between constructive internet use and subjective well-being, however, has not been explicitly assessed before. Thus, these findings provide meaningful insight into the health-related outcomes of constructive internet behavior.

Finally, the fifth hypothesis, which explored whether constructive internet use and health-related internet use predicted quality of life, health satisfaction, sleep satisfaction, and PA self-efficacy, was partially supported by the data. Only sleep satisfaction and PA self-efficacy were predicted by the independent variables, while no relationship was found between quality of life and health satisfaction. Both internet use for work/study and apps for relaxation were found to be predictors with a negative effect on satisfaction with sleep. Similar to the first hypothesis, the negative effect of internet use for work/study on satisfaction with sleep could be explained by excessive internet use, particularly before sleep, which may disturb sleeping habits and increase the risk of sleep deprivation (Billari et al., 2018). Using the internet during nighttime may be a significant mediator of this relationship. Also, Custers and Van den Bulck (2012) concluded that internet availability in one’s bedroom predicts a change in their sleeping schedule, with later bedtime and later rise time on weekdays, and later bedtime on weekends. The negative association of relaxation apps’ use with sleep satisfaction could be mediated by the purpose of using relaxation apps. For instance, if one uses relaxation apps to reduce anxiety or depression, the presence of such disorders may itself be a factor that reduces satisfaction with sleep. Although research has shown that relaxation and breathing techniques may promote behavioral change in sleeping habits and improve sleep disturbances (Sato, 2020), their full effects may only be achieved after several months of regular practice (Tsai, 2004).

A study conducted by Tsai (2004) found that relaxation techniques had no effect on sleep quality in healthy participants; however, they had a positive effect on anxiety in adults affected by cardiac disease. Thus, the presence of health impairments among participants might be a possible explanation for the negative relationship between relaxation apps and sleep satisfaction. The regression analyses revealed the use of apps for relaxation had a negative effect on PA self-efficacy, while using apps for movement/fitness had a positive effect. Again, the purpose of using relaxation apps could be a significant mediating factor. If participants use relaxation apps to treat insomnia, anxiety or depression, such disorders would be associated with less PA and thus lower PA self-efficacy. The positive effect of apps for movement/fitness on PA self-efficacy was expected. The reciprocal relationship between PA and PA self-efficacy has been further explained by McAuley and Blissmer (2000), who argue that PA self-efficacy is also an important outcome (Netz et al., 2005) in acute and regular exercise experiences.

### Limitations and Recommendations for Future Research

This study had a relatively large sample size and provided meaningful insight into predictors of subjective well-being that had not been assessed before; however, several limitations should be noted. First, there was a lack of prior research on constructive internet use and how it relates to subjective well-being and its components. The negative correlation between constructive internet use and satisfaction with sleep could be mediated by various variables that were not assessed in this study, such as level of stress, sleep disorders, using the internet for other purposes, daily schedule, etc. Similarly, the lack of correlation between constructive internet use and quality of life could be a result of other unassessed mediators (e.g., working conditions, personality traits, etc.) or undetected patterns from the correlational analysis.

Second, the lack of correlation between the use of health apps and quality of life could be attributed to the *t*-test as the chosen method of analysis. The independent samples *t*-test only represents the mean differences between the two groups (users and non-users of health apps). However, it does not represent a correlational or predictive relationship between the two variables. The effect of mediators such as the purpose of using health apps, health history and current health status of participants, and apps’ design and usability, needs to be thoroughly assessed as well. Third, the study uses self-reported data with limited reliability as it depends solely on the participants’ interpretation and introspective ability, which is prone to response bias (e.g., agreement bias or exaggeration).

Future research should focus on the underlying processes that moderate the relationship between constructive internet use and subjective well-being, and particular attention should be brought to the interaction between satisfaction with sleep and quality of life. Longitudinal studies should be conducted to examine the effect of using health apps regularly over a specific time period (e.g., 3–6 months). This would provide more accurate results on the impact of health apps on subjective well-being. Since the nature of the current study is correlational, further research should implement an experimental design with constructive internet use and regular use of health apps as controlled, independent variables. This would help establish a causal relationship between these factors and subjective well-being and its components.

### Conclusion

This study filled a gap in both the well-being literature and that of online behavior, as the relationship between constructive internet use and subjective well-being had not been assessed by researchers. This research has introduced a new perspective on constructive and healthy internet use, with the conservation of resources theory explaining gratifying internet behavior as a trigger for a well-being-related outcome gain spiral. These theoretical implications may comprise a relevant framework for the development of online well-being interventions.

Moreover, this study provides important practical implications for motivating people to engage in healthy behaviors, in particular physical activity, as a way to increase their PA self-efficacy, and their overall level of well-being.

## Supplementary Materials

For this article the following Supplementary Materials are available via PsychArchives (for access see the Index of Supplementary Materials below)

- Two appendices with independent samples *t*-test results

GuzaE.
GaoL.
LippkeS.
 (2022). Supplementary materials to "Internet behavior and satisfaction with sleep, health, quality of life and physical activity self-efficacy as components of subjective well-being: Findings from an online survey"
[Appendices]. PsychOpen. 10.23668/psycharchives.10011
PMC978073736605085

## Data Availability

Data is freely available at Supplementary Materials
